# Impact of receiving recorded mental health recovery narratives on quality of life in people experiencing non-psychosis mental health problems (NEON-O Trial): updated randomised controlled trial protocol

**DOI:** 10.1186/s13063-022-06027-z

**Published:** 2022-01-29

**Authors:** Stefan Rennick-Egglestone, Rachel Elliott, Chris Newby, Clare Robinson, Mike Slade

**Affiliations:** 1 School of Health Sciences, Institute of Mental Health, University of Nottingham Innovation Park, Triumph Road, Nottingham, NG7 2TU UK; 2 Division of Population Health, Health Services Research & Primary Care, University of Manchester, Oxford Road, Manchester, M13 9PL UK; 3 School of Medicine, Institute of Mental Health, University of Nottingham Innovation Park, Triumph Road, Nottingham, NG7 2TU UK; 4 Centre for Primary Care & Public Health, Pragmatic Clinical Trials Unit, Queen Mary University of London, 58 Turner St, London, E1 2AB UK

**Keywords:** Randomised controlled trial, Pragmatic trial, Recovery narratives, Recovery stories, Quality of life, MANSA, Mental health, Online trial, Open recruitment

## Abstract

**Background:**

Mental health recovery narratives are first-person lived experience accounts of recovery from mental health problems, which refer to events or actions over a period of time, and which include elements of adversity or struggle, and also self-defined or observable strengths, successes, or survival. Recorded recovery narratives are those presented in invariant form, including text, audio, or video. In a previous publication, we presented a protocol for three pragmatic trials of the Narrative Experiences Online (NEON) Intervention, a web application recommending recorded recovery narratives to participants. The aim of the definitive NEON Trial was to understand whether the NEON Intervention benefitted people with experience of psychosis. The aim of the smaller NEON-O and NEON-C trials was to evaluate the feasibility of conducting definitive trials of the NEON Intervention with people (1) experiencing non-psychosis mental health problems and (2) who informally care for others experiencing mental health problems.

An open recruitment strategy with a 60-week recruitment period was developed. Recruitment for the NEON Trial and NEON-O Trial targeted mental health service users and people not using mental health services. The NEON Trial recruited to time and target. The NEON-O Trial achieved its target in 10 weeks. Analysis considered by a Programme Steering Committee after the target was achieved demonstrated a definitive result could be obtained if the trial was adapted for recruitment to continue. The UK Health Research Authority approved all needed amendments following ethical review.

**Purpose of this article:**

To describe the decision-making process for amending the NEON-O Trial and to describe amendments made to the NEON-O Trial to enable a definitive result. The article describes amendments to the aims, objectives, design, power calculation, recruitment rate, process evaluation design, and informed consent documents. The extended NEON-O Trial adopts analysis principles previously specified for the NEON Trial. The article provides a model for other studies adapting feasibility trials into definitive trials.

**Trial registration:**

All trials prospectively registered. NEON Trial: ISRCTN11152837. Registered on 13th August 2018. NEON-C Trial: ISRCTN76355273. Registered on 9th January 2020. NEON-O Trial: ISRCTN63197153. Registered on 9th January 2020. The NEON-O Trial ISRCTN was updated when amendments were approved. Amendment details: NOSA2, 30th October 2020.

**Supplementary Information:**

The online version contains supplementary material available at 10.1186/s13063-022-06027-z.

## Background

Mental health recovery narratives have been defined as first-person lived experience accounts of recovery from mental health problems, which refer to events or actions over a period of time, and which include elements of adversity or struggle, and also self-defined strengths, successes, or survival [1, 2]. Recorded recovery narratives are those presented in invariant form, such as in text, audio, or video [3]. They are frequently grouped into named collections, sometimes presented online and sometimes in printed media [4, 5]. There are opportunities for integrating recorded recovery narratives into health service practices [6], and recovery narratives can describe post-traumatic growth and hence might inform trauma-informed practice [7].

The NEON (Narrative Experiences Online) study has developed the NEON Intervention, a web-application recommending items from the NEON Collection of recovery narratives to its users [8]. The change model underpinning the NEON Intervention is primarily transdiagnostic, based on empirical studies demonstrating a range of transdiagnostic benefits due to receiving mental health recovery narratives [3, 8–10]. In a previous publication, we have presented a protocol describing three pragmatic trials of the NEON Intervention which are being conducted by the NEON study, each with a different aim [11]. The aim of the definitive NEON Trial was to understand whether receiving online recorded recovery narratives through the NEON Intervention benefitted people with experience of psychosis. The aim of the smaller NEON-O and NEON-C trials was to evaluate the feasibility of conducting future definitive trials with people experiencing non-psychosis mental health problems and who care for others experiencing mental health problems respectively. In these three trials, the NEON Intervention and all trial procedures were delivered through a single shared web application hosted at https://recoverystories.uk. An online questionnaire shared between the three trials was used to determine participant eligibility and trial allocation.

An open recruitment strategy [12] with a 60-week planned recruitment period was adopted for all three trials. Recruitment for the NEON Trial and NEON-O Trial targeted both current and prior mental health service users, and also people who had never used mental health services. Recruitment work encompassed recruitment by clinical support officers in 11 secondary care mental health trusts, and also recruitment through a range of online mechanisms such as the placement of adverts on websites and the publication of large numbers of targeted social media posts. The latter was enabled at scale by the development of recruitment principles governing the design of ethically-appropriate recruitment messages. These principles were approved in advance by a research ethics committee [13].

The first enrolments to the NEON Trial and the NEON-O Trial were on 9th March 2020. The NEON Trial recruited to target within the 60-week recruitment period. The NEON-O Trial met its initial recruitment target of 100 participants within the first 10 weeks of the 60 week recruitment period. After consultation with a Programme Steering Committee (PSC), the sample size was subsequently amended to 350 participants to allow for a more accurate estimate of trial parameters if the NEON-O Trial was extended to produce a definitive result. Parameters considered were recruitment rate, retention rate, and distribution of the primary outcome.

The progress of the NEON trials as a whole was evaluated at a pre-planned PSC meeting on 7 July 2020. This was presented with recruitment, retention and interview evidence collected during the first three months of the NEON study. The PSC recommended that no major changes to trial procedures should be made. It also recommended that a review should be conducted of the NEON-O Trial once it met or came close to meeting its revised sample size so as to determine whether it should be extended into a definitive trial.

The review of the NEON-O Trial was conducted at a PSC meeting on 11th September 2020. Recruitment rates, retention rates, and the distribution of the primary outcome were reviewed. The PSC concluded it was feasible for the NEON-O Trial to produce a definitive result within the planned recruitment period if recruitment continued. A recommendation for a power calculation was agreed. A Notice of Substantial Amendment (NOSA) was subsequently submitted for approval by a National Health Service (NHS) ethics committee and the UK Health Research Authority (HRA). In developing this amendment, the selected approach was to align the aims and objectives of the NEON-O Trial with the NEON Trial. This approach allows a single Statistical Analysis Plan (SAP) to be devised to cover both trials. It allows the development of a single set of analysis scripts for use in both trials. Once approvals for the amendment were received, Principle Investigators at the 11 participating research sites were informed.

In addition to documenting the decision-making process for amending the NEON-O Trial as described above, the purpose of this article is to detail the amendments that were made to items in our published protocol to enable the NEON-O Trial to be extended to produce a definitive result. As well as establishing the effectiveness of the intervention for the NEON-O study population, amended protocol items enable cost-effectiveness to be established and a comprehensive process evaluation to be conducted [11]. Understanding the effectiveness and cost-effectiveness of receiving recorded mental health recovery narratives through the NEON Intervention for the populations selected for the NEON Trial and the NEON-O Trial will allow for transdiagnostic recommendations to be submitted to the National Institute for Clinical Excellence (NICE), and will contribute to an ongoing debate on the value of transdiagnostic approaches to mental health treatment [14]. A more comprehensive process evaluation will inform refinement of the existing trial change model with additional transdiagnostic and diagnosis-specific components.

## Updated protocol elements for the extended NEON-O Trial

The following amendments were made to the previously-published protocol [11].

### Aims

The amended aim of the NEON-O Trial is to understand whether receiving online recorded recovery narratives benefits people with experience of non-psychosis mental health problems. This aim was selected to match the aim for the NEON Trial.

### Primary objectives

In keeping with the NEON Trial, the amended primary objective for the NEON-O Trial is to evaluate the effectiveness of the NEON Intervention in improving quality of life at 1 year follow-up. The amended primary hypothesis is that compared to control group participants not receiving the NEON intervention during that year, intervention group participants who receive the NEON intervention will have a clinically-important and significant increase in quality of life one year later.

### Secondary objectives

In keeping with the NEON Trial, the amended secondary objectives for the NEON-O Trial are as follows:

1. To evaluate effectiveness in improving hope, empowerment, meaning in life, and reducing symptomatology
2. To evaluate the cost-effectiveness of the intervention compared with treatment as usual, from both a health and social care provider, and a societal perspective.
3. To understand how the intervention is used and experienced
4. To evaluate the trial change model
5. To evaluate the performance of the supervised machine-learning algorithm in producing a model that matches recovery narrative content to participants
6. To understand how the model trained by the machine-learning algorithm develops through the trial
7. To determine whether the effectiveness of the NEON intervention varies according to prior health-service usage by a participant.

### Exploratory objectives

In keeping with the NEON Trial, the amended exploratory objectives for the NEON-O Trial are as follows:

1. To identify potential predictors of outcome, to inform the design and analysis of future trials
2. To examine how the effect of the intervention varies over time and by dose

### Design

The amended NEON-O Trial is a randomised controlled trial with an economic and process evaluation. Participants who meet inclusion criteria will be individually randomised into one of two treatment groups (control group, intervention group) with an allocation ratio of 1:1. Follow-up is at one week, 12 weeks and 52 weeks after randomisation, with the primary end point at 52 weeks. Cost-effectiveness of the NEON Intervention will be established by calculating the costs of delivering the NEON Intervention, the impact on services costs of receiving the intervention, and the change in Quality-Adjusted Life Years (QALYs) due to receiving the intervention.

### Power calculation

The amended NEON-O Trial is powered on mean item score in the 12 subjective items defined in the Manchester Short Assessment of Quality of Life (MANSA) [15]. In keeping with the NEON Trial, the primary endpoint is a minimally clinically important difference in mean item score, defined as an improvement of 1 scale point in 3 out of 12 items at 1-year follow up in the intervention group relative to the control group [16]. The population for the NEON-O Trial is broadly defined, and hence the standard deviation of the MANSA index score for the study population was estimated from baseline data provided by the first 350 participants enrolled in the NEON-O Trial when initially configured as a feasibility trial. Attrition was estimated from missing data at week 12 follow-up, as this was considered an appropriate indicator of likely attrition at week 52 follow-up. A safety margin was incorporated as recommended by the PSC.

With this approach, a total sample size of 994 (497 participants per arm) was selected to provide 90% power to detect a minimally clinically-important effect size (Cohen’s *d*) of 0.27 (SD=0.94, power = 0.9, *p* = 0.05) allowing for 40% attrition. The analysable sample is 596 (298 participants per arm). The mean weekly recruitment rate was 16.6 participants per week for 60 weeks.

### Qualitative process evaluation data collection

In keeping with the NEON Trial, qualitative process evaluation data will be collected from the following groups:

1. Any trial participants who withdraw their consent for participation (the withdrawal of consent group) will be given the opportunity to tell the research team by email about how NEON can be improved. As these are no longer participating in the trial, they will not be interviewed but simply given the opportunity to provide feedback, which will be retained anonymously. Beyond clarifying any ambiguities, dialogue will not be entered into following receipt of any feedback, other than to thank the person for their feedback.
2. Intervention group participants will be offered the opportunity to take part in the process evaluation of the trial at the end of their one-year participation. Interviewing will continue with these groups until either theoretical saturation or 50 interviews per trial have been completed, whichever is sooner.
3. Up to 20 intervention group participants who only minimally used the intervention (low engagement group) will also be conducted. Sufficient interviews will be conducted to understand the perspective of people who may not have found the intervention useful.

### Informed consent documents

The Participant Information Sheet (PIS) was amended to indicate that NEON-O Trial participants will be paid for process evaluation interviews (Additional file 1). For parity, this amendment also extended payment to NEON-C Trial participants. The Informed Consent Form (ICF) was amended so that versioning information is correct (Additional file 2). Participants enrolled after approval of the amendment received the amended versions of the PIS and ICF. Participants enrolled before the amendment was approved were notified of the change when approached to take part in process evaluation interviews, and were informed that they did not need to accept payment (for example if payment might disrupt payment of state benefits).

### Analysis principles

A statistical analysis plan will be developed in advance of any analysis, and descriptive, clinical outcomes, exploratory and economic analyses will adopt the principles specified for the NEON Trial in our published protocol, including the specified significance threshold [11].

## Supplementary Information

**Additional file 1.** Amended Participant Information Sheet (PIS). Online PIS used for the NEON Trial, NEON-O Trial and NEON-C Trial.

**Additional file 2.** Amended Informed Consent Form (ICF). Online ICF used for the NEON Trial, NEON-O Trial and NEON-C Trial.

## Data Availability

Anonymous and pseudonymous elements of the datasets used and/or analysed during the study will be available on request from the study team before the NEON Study ends, although requests may be refused whilst research publications are being generated. After the NEON Study ends, anonymous and pseudonymous research data will be available from the study sponsor on reasonable request until the end of the retention period, but request may be refused if NEON study investigators are still generating research publications from this data. After the retention period, availability through the study sponsor or Chief Investigator may be provided at their discretion. Contact the study sponsor through Research@nottshc.nhs.uk.

## Abbreviations

HRA: Health research authority
ICF: Informed consent form
ISRCTN: International Standard Registered Clinical/soCial sTudy Number
MANSA: Manchester short assessment of quality of life
NEON: Narrative experiences online
NICE: National Institute for Clinical Excellence
NHS: National health service
NOSA: Notice of substantial amendment
PIS: Participant information sheet
QALY: Quality-Adjusted Life Year
