# Supplementary material for: Impact of receiving recorded mental health recovery narratives on quality of life in people experiencing non-psychosis mental health problems (NEON-O Trial): updated randomised controlled trial protocol
Source: Trials. 2022 Jan 29;23:90. doi: 10.1186/s13063-022-06027-z (PMC8800358; doi:10.1186/s13063-022-06027-z)
Supplement: Supplementary file 1 — Additional file 1. Amended Participant Information Sheet (PIS). Online PIS used for the NEON Trial, NEON-O Trial and NEON-C Trial. [file 13063_2022_6027_MOESM1_ESM.pdf]

Online Participant Information Sheet – [insert name of trial]

The following three paragraphs explain how the online PIS will appear to a potential participant. They will not be included in the PIS itself.

This is the online PIS for all three trials. The study logo and the study sponsor’s logo will appear at the top of the online implementation of the PIS, and are included above. Because this is a national online trial then the PIS will not be localised to the recruiting research site, and hence the research site logo will not be included. The names of the trials to be used in the PIS are NEON Trial, NEON-O Trial and NEON-C Trial.

The PIS consists of the following numbered items. Some of these items include text that will need to be specialised to the trial that the potential participant has been allocated to. and for these, the specialised text to use is included.

Each item in the PIS will initially be displayed with a title and a piece of brief text. The item number included in the text below is purely for reference, and will not be visible to the potential participant. Some items have additional text, which can be examined by the potential participant if these choose to do so. It will be clearly visible to the potential participant that additional text is available.

Item 1

Title: Information for people interested in <insert name of trial>

Brief text: Participant Information Sheet version 4.0. 13th October 2020.

We would like to invite you to take part in our research study. This participant information sheet will help you understand why the research is being done and what it would involve for you, if you wish to take part. You do not have to take part in this study; joining is entirely up to you.

Additional text: The reference number for this research study is 249015

Item 2

Title: What is <insert name of trial>?

Brief text: [insert name of trial] is a healthcare research study. It aims to understand whether reading, watching or listening to the stories of people who have recovered from mental health problems can help [people who have experienced what some call psychosis | people who have experienced mental health problems | people who have cared for others with mental health problems]. [insert name of trial] is taking place as part of the NEON research study, which runs until September 2022.

Item 3

Title: What are recovery stories?

Brief text: A recovery story is any story that that describes one or more periods of adversity that relate to mental health problems, and also one or more periods of success, strength or survival that relate to these.

Item 4

Title: How would I access recovery stories?

Brief text: Through the NEON Intervention, a website which gives access to hundreds of recovery stories. It can be used on a smartphone or a computer. The NEON Intervention tries to match you to stories which might help you, using information about yourself that you provide. It also allows you to browse stories based on a range of categories, or to return to stories that you have previously accessed. Stories are presented in a mixture of text, video, audio and images. You can choose to hide particular formats of story if you wish, and any type of content that causes you distress.

Item 5

Title: What will happen next if I decide to take part in [insert name of trial]?

Brief text: You will be asked to fill out an online consent form, through which you must provide a working email address. You don't have to provide your name. You will be asked to provide a password for an online account. You will also be asked for some basic information about yourself using an online survey. You will be asked to fill out the online survey again after 1 week, 12 weeks and 52 weeks. This is an online trial; you will not be expected to meet with researchers or to visit treatment centres.

To test whether accessing recovery stories through the NEON Intervention can significantly improve user's quality of life, we need to compare people who receive immediate access with similar people who do not receive immediate access. So, using a list of random numbers, you will be selected to either receive immediate access to the NEON Intervention, or to receive access after one year.

In the first few months of the trial, and after taking part for one year, you might be asked to take part in an interview by telephone or secure video conference to help us understand what it feels like to take part. You don't have to take part in an interview if you don't want to, and you can continue participating in the trial even if you refuse. If you are interviewed, then we will record the sound of your interview and store the recording. We may also transcribe it. If we do, we will remove any information that identifies you from the transcripts.

Item 6

Title: If I get access to the NEON Intervention, how much do I have to use it?

Brief text: You can use it as little or as much as you want until the end of the trial. It's up to you. The end of the trial is currently planned for 30th April 2022.

Item 7

Title: If I don't get immediate access to the NEON Intervention, do I still have to continue with [insert name of trial]?

Brief text: No, as you can withdraw at any point, but your continued involvement would be appreciated and your responses to the online questionnaires are important for the Trial.

Item 8

Title: Do I have to take part in [insert name of trial]?

Brief text: No, and if you decide not to participate this will not affect any medical or social care you are receiving.

Item 9

Title: What will I receive for taking part?

Brief text: [Text in this paragraph is displayed for the NEON Trial only]You can claim a £20 online voucher for completing each round of our online survey. If you choose to claim a voucher, then your email address will be passed to a study administrator who will send it to you. We aim to send vouchers within a week of claim; but payment can be delayed or cancelled if we detect unusual patterns (such as many vouchers being claimed from the same computer).

[Text in this paragraph is displayed for all trials]If you are asked to take part in an interview, then you will be offered £20, which you can be paid through an online voucher or by submitting a claims form. We aim to send vouchers within a week, and payment by claims form can take up to 6 weeks.

Item 10

Title: What are the possible benefits of taking part?

Brief text: Our research has identified a range of ways in which recovery stories can benefit people. They can help you feel more hopeful, connected, or more appreciative of your own life. You can learn about strategies for recovery. They might help you decide that you need to look for help. They can expose you to people and ideas that you might not find in your everyday life. NEON is a research study, and we cannot guarantee that it will benefit you. If you are receiving treatment, you should consult your healthcare professional before making any changes to your treatment.

Item 11

Title: What are the possible harms caused by taking part?

Brief text: Our research has shown some ways in which recovery stories can harm people. Learning about the difficulties of others might make you feel more pessimistic or emotionally burdened. Some people can feel disconnected or inadequate if they read a story and feel that they can't match what the person telling it has achieved. Some people experience the release of uncomfortable emotions after receiving stories. There is some evidence that reading stories describing harmful behaviours can create a risk of copying those behaviours, especially for people with similar experiences.

To help us monitor any harmful effects of the study, we have provided an online form which you can use to tell us about incidents of a serious nature. We understand these can be distressing and information on help and support organisations or services will be available to you if you need it, through a page in the intervention.

Item 12

Title: Will you contact me during [insert name of trial]?

Brief text: Yes, by email, or by SMS or telephone if you have chosen to provide a mobile phone number. We might contact you to remind you to complete online surveys or to let you know about new recovery stories. You will only be contacted by the University of Nottingham (who lead the trial) and by Nottinghamshire Healthcare Trust (the study Sponsor), and only then in relation to this study.

Item 13

Title: Who is organising [insert name of trial]?

Brief text: [insert name of trial] is led by Professor Mike Slade at the University of Nottingham. The NEON study is sponsored by Nottinghamshire Healthcare NHS Foundation Trust, and [insert name of trial] is overseen by the Pragmatic Clinical Trials Unit at Queen Mary University of London.

Item 14

Title: Who has approved [insert name of trial]?

Brief text: The [insert ethics committee name], the Health Research Authority, the study sponsor and the Pragmatic Clinical Trials Unit.

Item 15

Title: Can I withdraw from [insert name of trial]?

Brief text: You can stop being part of the study at any time, without giving a reason. We will keep information about you that we already have, other than any information that can identify you which will be deleted.

Item 16

Title: Will my taking part in [insert name of trial] be kept confidential?

Brief text: Any information that might identify you will be held confidentially. As safety is our priority, confidentiality may be breached if any of your responses suggests that you or others are at risk of harming yourself or others, or that you or others have engaged in criminal activities that require notification to authority. Decisions to breach confidentiality will be made in collaboration with the Chief Investigator, and ideally with yourself.

Item 17

Title: How will we use information provided by you?

Brief text: In this research study we will use information provided by you. We will only use information that we need for the research study.

The study sponsor has overall responsibility for your information (they are the ‘data controller’). You can contact them to find out more about how your information is managed: research@nottshc.nhs.uk. Everyone involved in this study will keep your identifiable information safe and secure. We will follow all privacy rules.. People will use this information to either do the research or to check your records to make sure that the research is being done properly.

Information will initially be collected on a web-server operated by DRT Software, a software contractor. Information will be copied to research servers run by the University of Nottingham. DRT and the University of Nottingham will delete any information that identifies you by the end of the NEON study (currently September 2022). The study sponsor has approved the arrangements with DRT Software and the University and Nottingham, and contracts are in place to ensure that information is used appropriately. Information that cannot identify you may be shared with other researchers.

At the end of the study we will save some of this information in case we need to check it, and for future research. We will make sure no-one can work out who you are from the reports we write. Other researchers might be granted access to those parts of the research data that cannot identify you, and some of it may be used in research publications. After the NEON study has closed, only information that cannot identify you will be retained by the study sponsor.

Item 18

Title: How can I find out more about how my information is used?

Brief text: You can find out more about how we use your information at www.hra.nhs.uk/information-about-patients/, by reading our leaflet at www.hra.nhs.uk/patientdataandresearch, by contacting the research team using neon@nottingham.ac.uk or 07973 841271, or by contacting the study sponsor using research@nottshc.nhs.uk.

Item 19

Title: I have some more questions about [insert name of trial]. Who can I contact?

Brief text: You can contact the research team using neon@nottingham.ac.uk, or you can leave a voice message or send a text to 07973 841271. We aim to respond within three working days.

Item 20

Title: How can I learn about the results of [insert name of trial]?

Brief text: All results will be published in a format that is openly accessible to the public. You will be able to find all of our study publications on the study website: http://www.researchintorecovery.com/neon. The results of [insert name of trial] will not be published until 2022 at least. Participants will never be named in publications.

Item 21

Title: Who is funding the NEON study and [insert name of trial]?

Brief text: The National Institute for Health Research

Additional text: The grant number for the NEON study is RP-PG-0615-20016, and it is funded under the Programme Grants for Applied Research funding scheme.

Item 22

Title: What should I do if I feel upset or distressed whilst using the NEON Intervention?

Brief text: If you choose to sign up to NEON, then you'll have access to lots of ideas for what to do. Click on the page called "I'm feeling upset" as a starting point. This page has been developed by people with their own experiences of mental health distress. It includes ideas for self-management of distress, links to online peers support services, and details of support that can be accessed through the NHS or charities.

Item 23

Title: Something has gone wrong – who should I contact?

Brief text: If you are experiencing technical problems with our online system, please contact neon@nottingham.ac.uk. If you are worried about how the study is being run, please contact the study coordinator using <insert study coordinator email address>. Alternatively, you can contact the study sponsor using <insert study sponsor email address>, or contact your local Patient Advice and Liaison service, whose contact details can be found on the Internet.
