# Supplementary material for: Impact of receiving recorded mental health recovery narratives on quality of life in people experiencing non-psychosis mental health problems (NEON-O Trial): updated randomised controlled trial protocol
Source: Trials. 2022 Jan 29;23:90. doi: 10.1186/s13063-022-06027-z (PMC8800358; doi:10.1186/s13063-022-06027-z)
Supplement: Supplementary file 2 — Additional file 2. Amended Informed Consent Form (ICF). Online ICF used for the NEON Trial, NEON-O Trial and NEON-C Trial. [file 13063_2022_6027_MOESM2_ESM.pdf]

Nottinghamshire Healthcare NHS
NHS Foundation Trust

Informed Consent Form - <insert trial name>
Version 4.0. 13th October 2020.

If you have any questions about this form, you can contact the research team through neon@nottingham.ac.uk or can leave a voice message or send a text to 07973 841271.

To provide your consent to take part in the NEON study, **please select "Yes"** against questions 1-7.

[*Note to REC: Each of the boxes presents a drop-down menu which defaults to the value "No", and potential participants must change this to "Yes"*]

1. I have read and understand the online participant information sheet (PIS) dated version 4.0. 13th October 2020.	1
2. I understand that my participation is voluntary, and that I can withdraw at any time without giving any reason, without my care or legal rights being affected.	2
3. I understand that my data will be held by DRT Software, Nottingham University and Nottinghamshire Healthcare NHS Foundation Trust	3
4. I understand that any contact details that I provide can only be used to contact me about the study, and for no other reason	4
5. I understand that my data will be accessed by the study team, and may be audited by Nottinghamshire Healthcare NHS Foundation Trust and the Pragmatic Clinical Trials Unit at Queen Mary University of London	5
6. I consent to the use of an anonymised version of information collected about me in this and other research studies.	6
7. I agree to take part in this research study	7
8. OPTIONAL: I would like to take part in an interview about the trial (by telephone or video-conference). I understand this is optional and does not affect my trial participation.	8

To create a login for you, we need an email address. Please enter this below, and we'll send an email with a link to click, to check it is working.

We don't need to know your name for this study. If you don't want us to know your name, you could use an email address which doesn't include your name.

…………………………………

We can also use text messages to contact you about the study. If you would like to provide a mobile number, please enter it below.

…………………………………

<Provide two buttons: "I agree to take part in the study" or "I do not wish to take part">
